# Evaluation of the efficacy and safety of ionic liquids containing ketoconazole in patients with tinea pedis: A randomized controlled clinical trial

**DOI:** 10.1002/btm2.10463

**Published:** 2022-12-02

**Authors:** Xiying Wu, Min Shen, Huan Wang, Xue He, Jingwen Tan, Ruiping Wang, Lianjuan Yang, Hong Yang, Jianping Qi, Zhongjian Chen, Quangang Zhu

**Affiliations:** ^1^ Shanghai Skin Disease Hospital Tongji University School of Medicine Shanghai China; ^2^ School of Pharmacy Fudan University Shanghai China; ^3^ School of Pharmacy Naval Medical University Shanghai China

**Keywords:** Daktarin®, ionic liquids, ketoconazole, randomized controlled trial, tinea pedis

## Abstract

Ionic liquids (ILs) loading ketoconazole (KCZ) have shown better efficacy on rats with tinea pedis than the marketed Daktarin® but clinical studies are still lacking. In this study, we described the clinical translation of ILs containing KCZ (KCZ‐ILs) from the lab into the clinic and evaluated the efficacy and safety of KCZ‐ILs in patients with tinea pedis. Thirty‐six enrolled participants were randomized to receive either KCZ‐ILs (KCZ, 4.72 mg/g) or Daktarin® (control group; KCZ, 20 mg/g) topically twice daily, making the lesion be covered with a thin layer of medication. The randomized controlled trial lasted for 8 weeks including 4 weeks of intervention and 4 weeks of follow‐up. Primary efficacy outcome was the proportion of treatment success responders, defined as patients achieving negative mycological result and ≥60% relative reduction in total clinical symptom score (TSS) from baseline at week 4. Secondary outcomes mainly for evaluating the relapse of disease included the proportion of treatment success individuals at week 8 and fungal recurrence rate at weeks 2, 3, 4, and 8. After 4 weeks of medication, 47.06% of the KCZ‐ILs subjects were treatment successes compared with only 25.00% of those using Daktarin®. Throughout the trial period, KCZ‐ILs induced a significantly lower recurrence rate (52.94%) than that of control patients (68.75%). Furthermore, KCZ‐ILs were found to be safe and well‐tolerated. In conclusion, ILs loading only 1/4 KCZ dose of Daktarin® showed a better efficacy and safety profile in the management of tinea pedis, creating a new opportunity for the treatment of skin diseases caused by fungal infection and is worthy of clinical application.

## 1 INTRODUCTION

Tinea pedis is a common superficial fungal infectious skin disease that exists worldwide.^1^ *Trichophyton rubrum* and *Trichophyton interdigitale* are reported as two major aetiological dermatophytes responsible for high prevalence and recurrence rates.^2^ Although tinea pedis is not fatal, patients have disproportionate itching, pain, and emotional distress, resulting in a significant decline in quality of life.

Ketoconazole (KCZ), an imidazole antifungal agent, is effective for both superficial and deep fungal infections.^3^, ^4^ Various reported serious side‐effects including liver toxicity, acute renal failure, and dermatological complaints greatly limit the oral administration of KCZ.^5^, ^6^, ^7^ In clinical practice, topical preparations containing KCZ have been shown to be helpful in the treatment of tinea pedis,^8^ tinea capitis, tinea corporis,^9^ tinea nigra,^10^ and pityriasis capitis.^11^ However, the commonly used 2% KCZ cream was usually prescribed under mild and dramatically improved symptoms,^12^, ^13^ or for combined drug administration.^9^ In essence, high octanol–water partition coefficient (logP, 4.31), poor solubility (40 μg/ml), and high affinity for keratinized tissues of KCZ lead to the poor transdermal effectiveness and bioavailability, hindering its usage in clinic.^14^, ^15^ Despite the availability of several effective KCZ‐loaded agents in the topical drug arena, their therapeutic outcome is less than optimal, calling for the development of innovative drug delivery systems.

Ionic liquids (ILs) hold great promise to be optimal active pharmaceutical ingredients or drug carriers owing to enhanced drug solubility, bioavailability, and superior antifungal efficacy.^16^, ^17^, ^18^, ^19^, ^20^ Recently, the IL of choline geranate ([Ch][Ger]) has highlighted the potential for effective treatment of tinea pedis. Specifically, our previous study showed [Ch][Ger] had excellent inhibitory activity against *T. Interdigitale*, a major aetiological dermatophyte of tinea pedis, as well as a significant synergistic antimicrobial effect with KCZ.^2^, ^21^ Further, ILs have remarkable permeability, suggesting the ability to clear the dermatophytes in skin more thoroughly.^22^ Moreover, the [Ch][Ger]‐based formulation in our preliminary research had better performance in treating rats with tinea penis than the marketed Daktarin®.^21^ According to these encouraging results, clinical studies of topical ILs‐based formulations for treating microbial infection are worth investigating.

Translation of the formulations from bench to bedside is of great significance, which could be facilitated by scale‐up, characterization, stability, mode of action, dosing, preclinical toxicology in animals, and finally, human studies on efficacy and safety.^22^ Following the first example of clinical translation of ILs technology for the treatment of dermatological conditions in 2020,^22^ herein, we demonstrated a small‐scale (36 patients enrolled) but successful clinical translation case of KCZ‐ILs, a modified aqueous solution of our previously published ILs‐based formulation.^21^ In this trial, the efficacy and safety of KCZ‐ILs and Daktarin® in patients with tinea pedis were studied systematically, providing a reference to guide more ILs‐based clinical transformation in future.

## 2 RESULTS

### 2.1 Translational aspects of ILs

To pave the way for ILs from bench to bed, scale‐up, characterization, stability, and dosing of formulations were studied here. The active ingredient in KCZ‐ILs, [Ch][Ger] (Figure S1), was synthesized at a kilogram scale using the same process as that at a laboratory scale of several grams. The resulting [Ch][Ger] is transparent at room temperature. No obvious impurities were observed during scale‐up via ^1^H‐NMR analysis (Figure S2).

KCZ‐ILs were manufactured by mixing the [Ch][Ger] with Tween‐80 and glycerin as co‐solvent, KCZ (Figure S1) as the antifungal drug and water as a dilution. In the Fourier transform infrared (FT‐IR) spectra (Figure S3), [Ch][Ger] exhibited C=O and —OH absorption bands at 1644 and 3233 cm^−1^, respectively. In case of KCZ‐ILs, the broad ‐OH peak from the [Ch][Ger] shifted to a higher wavenumber of 3345 cm^−1^, suggesting the change of intermolecular O—H⋯O hydrogen bonds. Besides, the vibration peaks of C—H changed from 2915 to 2922 cm^−1^, which indicated specific interactions in KCZ‐ILs. Additionally, it has been detected that [Ch][Ger] in KCZ‐ILs or alone dissociated partially since the peaks at 1689–1690 and 2967 cm^−1^ attributing to C=O and —OH stretch of carboxylic acid were observed.^22^ This system yields micelles of 11 nm in diameter determined by dynamic light scattering (DSL, Figure S4), which is similar to those found in other ILs‐based micelles.^23^

The packaging was performed in brown glass bottles. Due to the absence of covalent bonds in [Ch][Ger], the content of geranic acid and KCZ were determined with high‐performance liquid chromatography (HPLC) to reflect the signature of KCZ‐ILs. After 14 months, KCZ‐ILs demonstrated minimal change in stability with 2.9% loss of KCZ and 3.6% loss of geranic acid, respectively (Figure S5). Although an impurity peak with content of 7.9% appeared in the chromatographic profile of KCZ‐ILs, it did not interfere with the determination of key constituents. Additionally, no significant variation in the physical appearance was observed, suggesting that KCZ‐ILs can be well preserved for a period of at least 14 months.

**FIGURE 1:**
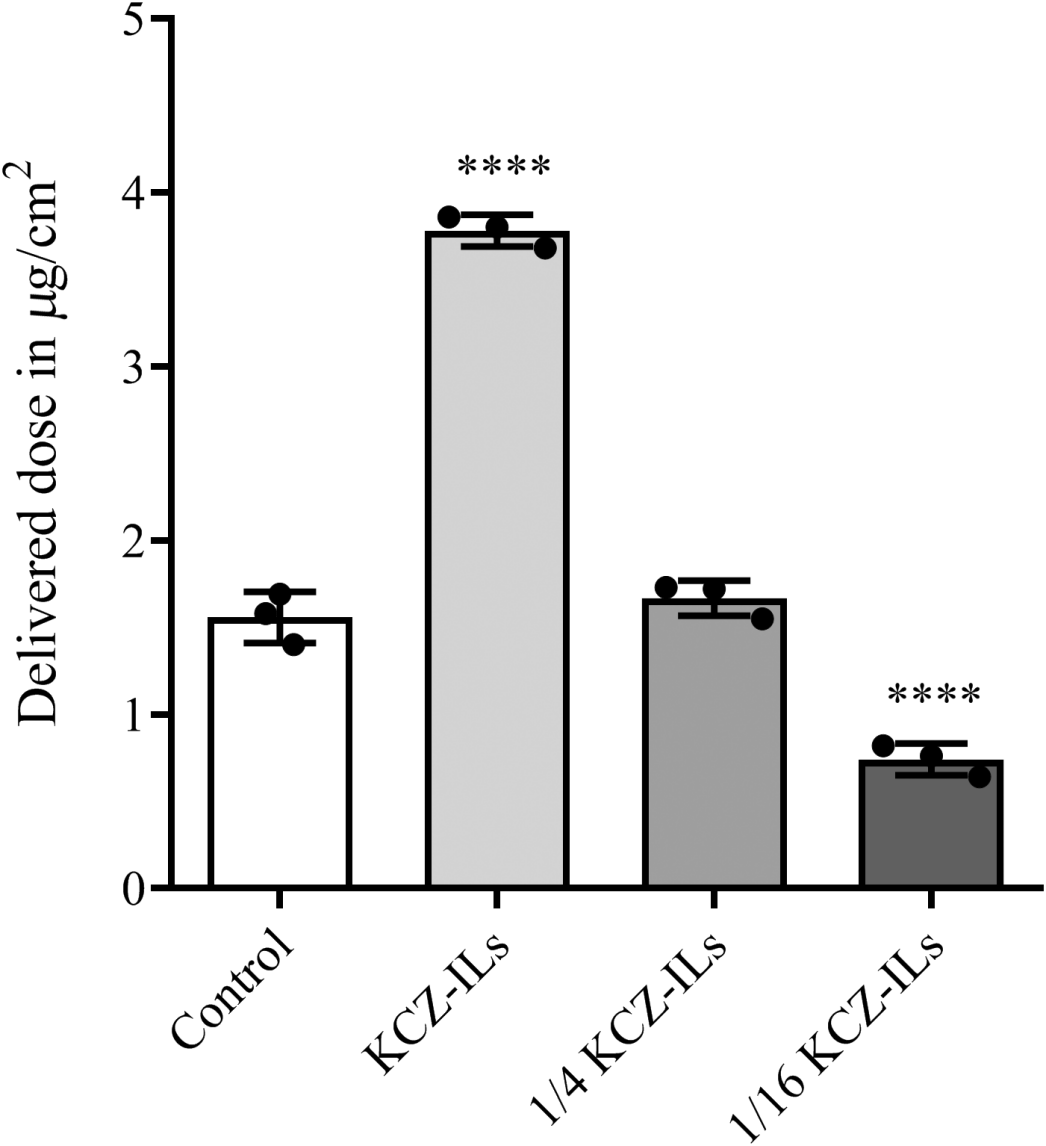
The amount of KCZ delivered into skin after treatment of various formulations. Data are mean ± SD (*n* = 3), statistics by one‐way ANOVA and ****p < 0.0001 compared with Daktarin® (Control).

Dosing was determined by the in vitro permeation test. The permeability of KCZ in ILs‐based aqueous solutions was assessed to determine the optimal formulation for the following clinical study. Among them, 1/4 and 1/16 KCZ‐ILs were obtained by diluting the KCZ‐ILs with water 4 and 16 times, respectively. As shown in Figure 1, KCZ‐ILs showed the optimal enhanced ability with the cumulative transdermal volume of 3.78 ± 0.09 μg/cm^2^, followed by those of 1/4 KCZ‐ILs (1.67 ± 0.10 μg/cm^2^) and 1/16 KCZ‐ILs (0.74 ± 0.09 μg/cm^2^) groups. Notably, the differences between the KCZ‐ILs, 1/16 KCZ‐ILs groups and the control group (1.55 ± 0.15 μg/cm^2^) were significant (*p* < 0.0001) while the 1/4 KCZ‐ILs group and the control group demonstrated evenly matched effect. In consideration of the low adhesion of aqueous solution, the KCZ‐ILs that facilitated the penetration of KCZ into the skin by over twofold compared to the control group were selected for administration.

### 2.2 Study population

**FIGURE 2:**
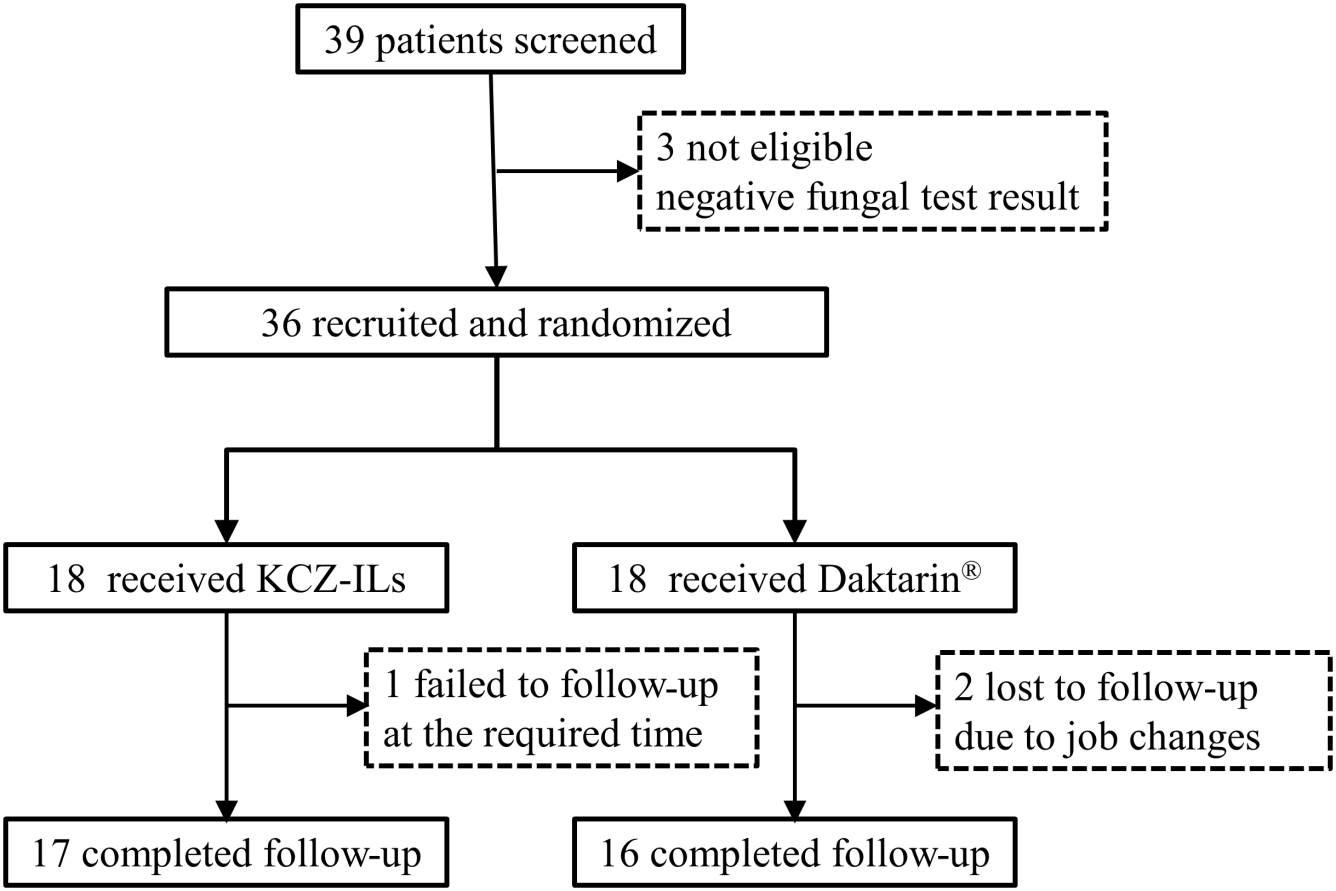
CONSORT diagram of the participants

A total of 39 patients underwent screening and 36 satisfying inclusion and exclusion criteria were enrolled. Three patients were lost in follow‐up because of their job changes or unpunctuality during the study period. Of the 33 complete evaluable patients, 17 were treated with KCZ‐ILs and 16 with Daktarin®. The CONSORT diagram of the participants is given in Figure 2. At the end of the treatment, adherence to the trial regimen in the KCZ‐ILs and Daktarin® groups was 96.4% and 98.4%, respectively.

Among the 17 cases in the experimental group, there were nine males and eight females with the age of 31–59 years old, and the average total clinical symptom score (TSS) of 8.76 (SD, 2.14). In the control group, there were eight males and eight females, whose ages range from 32 to 59 years old, and their average TSS was 9.44 (SD, 3.18). A few patients (4/33, 12%) had associated comorbidities such as hypertension (2/33, 6%), diabetes (1/33, 3%), or both (1/33, 3%). Besides the body‐mass index, the characteristics of the patients at baseline did not differ significantly between the two groups (Table 1).

**TABLE 1:** Demographic and clinical characteristics of the patients at baseline

Variable	KCZ‐ILs (*n* = 17)	Control (*n* = 16)
Age, mean (SD), years	48.88 (9.46)	49.13 (8.15)
Sex, *n* (%)		
Female	8 (47.06)	8 (50.00)
Male	9 (52.94)	8 (50.00)
Body‐mass index, mean (SD), kg/m^2^	24.09 (2.58)	24.47 (2.93)
Body‐mass index category, *n* (%)		
<25 kg/m^2^	10 (58.82)	11 (68.75)
25 ~ 30 kg/m^2^	6 (35.29)	4 (25.00)
>30 kg/m^2^	1 (5.88)	1 (6.25)
TSS, mean (SD)	8.76 (2.14)	9.44 (3.18)
Comorbidity,^a^ *n* (%)	2 (11.76)	2 (12.50)

^a^ Comorbidities were hypertension (*n* = 2, one in each group), diabetes (*n* = 1, in KCZ‐ILs group) or both (*n* = 1, in the control group).

### 2.3 Total clinical symptom score

**FIGURE 3:**
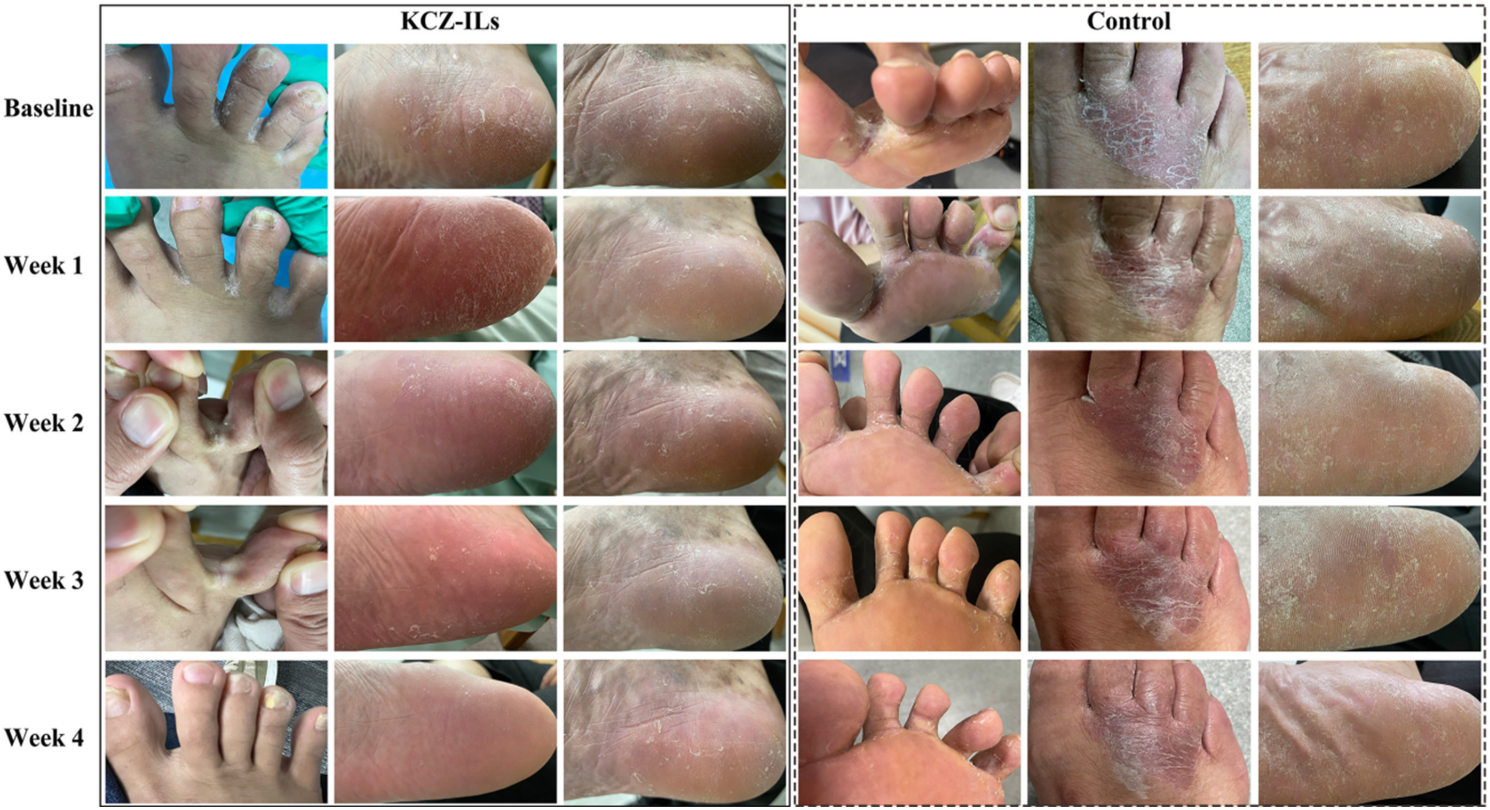
Representative photographs show the clinical course of patients with tinea pedis treated with KCZ‐ILs and Daktarin®. The clinical symptoms significantly improved with time in KCZ‐ILs group.

The change in TSS after 4 weeks of treatment was −4.94 (2.51) (*p* < 0.001) in the KCZ‐ILs group and −4.44 (2.34) (*p* < 0.001) in the Daktarin® group. The symptoms began to improve after only 1 week of treatment, and the effect lasted for the whole study period which could be visualized directly from representative photographs of patients with TSS ranged from 8 to 12 (Figure 3). The difference in TSS between the two groups was statistically significant (F = 58.58, *p* < 0.001), mainly contributed by weeks 2 and 3 (Table S2).

**FIGURE 4:**
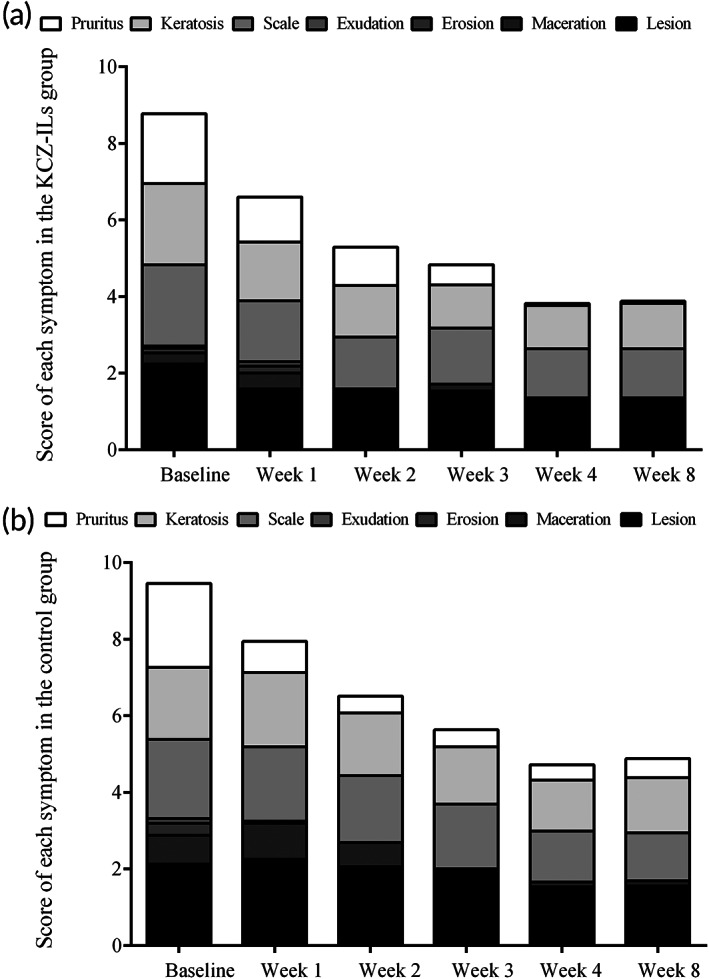
The scores of clinical symptoms in the KCZ‐ILs and control groups at each visit. Symptoms were grouped into seven categories and the severe score (0, none; 1, mild; 2, moderate; 3, severe) of any symptom in the specific category was used for the participants.

In order to clarify the specific effect of both formulations, seven signs including lesion size, maceration degree, erosion area, exudation degree, scale, keratosis, and pruritus, directly attributable to tinea pedis at the lesion site were evaluated. The results showed that KCZ‐ILs had a significant improvement on lesion area, scaling, keratinization, and pruritus while Daktarin® tended to relieve scaling and pruritus symptoms (Figure 4, Tables S3 and S4).

### 2.4 Mycological eradication rates

Mycological eradication rates evaluated by direct microscopic examination were presented in Table 2. Compared to baseline, the fungal eradication rate of KCZ‐ILs was 41.18% (*p* < 0.05) in week 1, 64.71% (*p* < 0.05) in weeks 2–3. At the end of treatment, the clearance rate reached 76.47% with only four positive patients left. In contrast, the fungi clearance rate of Daktarin® remained the same at 50.00% in weeks 1–3 and reached 56.25% in week 4.

**TABLE 2:** Mycological results of two groups

Variables	KCZ‐ILs (*n* = 17)	Control (*n* = 16)	*p* value^a^
Baseline, *n* (%)			
Positive	17 (100.00)	16 (100.00)	1.000
Negative	0 (0)	0 (0)	
Week 1, *n* (%)			
Positive	10 (58.82)	8 (50.00)	0.732
Negative	7 (41.18)	8 (50.00)	
*p* value^b^	0.002	0.152	
Week 2, *n* (%)			
Positive	6 (35.29)	8 (50.00)	0.491
Negative	11 (64.71)	8 (50.00)	
*p* value^b^	0.031	0.152	
Negative to positive^c^	2 (28.75)	3 (37.50)	0.325
Week 3, *n* (%)			
Positive	6 (35.29)	8 (50.00)	0.491
Negative	11 (64.71)	8 (50.00)	
*p* value^b^	0.031	0.152	
Negative to positive^c^	1 (9.09)	3 (37.50)	<0.001
Week 4, *n* (%)			
Positive	4 (23.53)	7 (43.75)	0.282
Negative	13 (76.47)	9 (56.25)	
*p* value^b^	0.125	0.230	
Negative to positive^c^	2 (18.18)	3 (37.50)	0.008
Week 8, *n* (%)			
Positive	7 (41.18)	8 (50.00)	0.732
Negative	10 (58.82)	8 (50.00)	
*p* value^b^	0.016	0.152	
Negative to positive^c^	4 (30.77)	2 (22.22)	0.216

^a^ Differences between the KCZ‐ILs group and the control group.

^b^ Differences compared to baseline in the KCZ‐ILs group or the control group.

^c^ The proportion of patients changed from negative to positive was calculated as the ratio of the number of new negative to positive cases to the total number of negative cases in the previous period.

Fungal recurrence cases appeared from the second week of dosing. The conversion rate of mycological negative to positive was the lowest at 9.09% at the third week of KCZ‐ILs dosing, and increased either by continuing the treatment for 1 week or by stopping it. In the control group, negative conversion rate remained at 37.5% from the second week to the fourth week of treatment, and unexpectedly dropped slightly after discontinuation. Throughout the trial period, there were nine (52.94%) fungal negative cases changed to be positive for KCZ‐ILs, compared with 11 (68.75%) of 16 for Daktarin®.

### 2.5 Overall efficacy outcomes

As the primary efficacy variable, overall efficacy was derived by integrating with mycological results and TSS improvement. A four‐grade scale (cured, markedly effective, effective, and ineffective) with proper modification of previously published categories was used in this study.^24^ Approximately 53% of patients achieved efficacy, 41% attained markedly efficacy, and 6% achieved a complete cure in the KCZ‐ILs group, compared to 19% for inefficacy, 56% for efficacy, 19% for markedly efficacy, and 6% for complete cure in the control group. The primary efficacy outcome was the proportion of treatment success (cured and markedly effective) responders. As shown in Table 3 and Figure S6, the treatment success rate of KCZ‐ILs was 47.06% while that of Daktarin® was only 25.00%, reflecting a significant difference (*p* = 0.010) in favor of KCZ‐ILs at week 4. Secondary efficacy outcomes were mainly for evaluating the relapse of disease. The proportion of treatment success individuals at endpoint, 4 weeks post‐treatment, was 41.17% for KCZ‐ILs compared to 31.25% for control subjects.

**TABLE 3:** Overall efficacy of two groups at weeks 4 and 8

Overall efficacy	KCZ‐ILs (*n* = 17)	Control (*n* = 16)
Week 4, *n* (%)		
Cured^a^	1 (5.88)	1 (6.25)
Markedly effective^b^	7 (41.18)	3 (18.75)
Effective^c^	9 (52.94)	9 (56.25)
Ineffective^d^	0 (0)	3 (18.75)
Treatment success^e^	8 (47.06)	4 (25.00)
Week 8, *n* (%)		
Cured	2 (11.76)	2 (12.50)
Markedly effective	5 (29.41)	3 (18.75)
Effective	9 (52.94)	10 (62.50)
Ineffective	1 (5.88)	1 (6.25)
Treatment success	7 (41.17)	5 (31.25)

^a^ Cured: negative mycological result plus clinical cure indicated by TSS.

^b^ Markedly effective: negative mycological result plus 60% ~ 99% clinical improvement.

^c^ Effective: negative or positive mycological result plus 20% ~ 59% clinical improvement.

^d^ Ineffective: positive mycological result plus less than 20% clinical improvement.

^e^ Treatment success incorporates the cured and markedly effective cases.

### 2.6 Safety results

No patients applying the KCZ‐ILs to lesion sites reported adverse reactions. One patient (6.25%) in the control group experienced transient mild erythema in the lesions 1 week after application of the drug, which did not affect the continuation of treatment and the determination of efficacy.

## 3 DISCUSSION

Clinical trials are conducted in humans to test new treatments, which are typically evaluated over several phases to determine their efficacy and safety. Phase 1 trials focus on the safety and tolerability, generally with a small number of healthy participants (e.g., 20–80). Phase 2 trials are the first time that new treatments are taken in target patients to observe the preliminary efficacy and safety, and to assess whether they have meaningful benefits to warrant further investigation in phase 3 trials. As phase 3 trials are usually multicenter, large‐scale, and long‐period trials, phase 2 trials acting as pioneers are of great significance.^25^ It is now established that the sample size of phase 2 trials ranges from tens to hundreds, of which a few hundred is more common.^25^ With reference to phase 2 trials, this study was designed to be a randomized controlled clinical trial with a small sample size. Herein, a sample size of 36 enrolled patients was determined by calculation, achieving 90% power and accounting for 20% dropout. Although the small sample size trials may not have enough statistical certainty to make decisive evaluations between new and conventional treatments, this cost control design can quickly provide more meaningful data for promising new treatments to enter further research phase.

ILs have shown potential for applications as antimicrobials. The choline‐based ILs, especially those synthesized with choline bicarbonate and geranic acid at ratios of 1:1 and 1:2, have been reported to possess prominent skin permeability and antimicrobial activity.^21^, ^22^, ^26^, ^27^ Moreover, the interactions between ILs and cell membranes opened a research avenue for ILs and antibiotics combinations to combat infections. A panel of imidazole‐based ILs combined with small‐molecule antibiotics were found to exhibit synergistic antimicrobial effects on both Gram‐positive than Gram‐negative bacterias.^28^, ^29^ Given these encouraging results, clinical studies of topical ILs‐based formulations for treating microbial infection are worth exploring. [Ch][Ger] was synthesized by a one‐step salt metathesis reaction and the scale‐up at a kilogram level proved to be feasible. The KCZ‐ILs formulation containing KCZ and [Ch][Ger] exhibited excellent stability under long‐term storage conditions. Additionally, KCZ‐loaded [Ch][Ger] used in this formulation can penetrate into deep skin, thus exerting strong antifungal activity.^21^ As 2% KCZ cream has been commonly used in many countries for the therapy of tinea pedis,^8^ Daktarin® (KCZ, 20 mg/g) was considered as a standard of treatment for a direct comparison with the KCZ‐ILs formulation in which the concentration of KCZ was 4.72 mg/g, about 1/4 of the control group. The efficacy in patients with tinea pedis was compared by mycological and TSS improvement, as well as the derived overall efficacy.

TSS was computed as the sum of seven signs attributable to tinea pedis at the lesion site where each sign score was rated on a scale of 0 (not present) to 3 (severe).^30^ The mean TSS changes (SD) after 4 weeks of treatment showed significant improvement from baseline in KCZ‐ILs (−4.94 [2.51], *p* < 0.001) and control groups (−4.44 [2.34], *p* < 0.001). Both formulations began to relieve the symptoms remarkably after only 1 week of dosing, and the effect lasted for the whole trial period. In particularly, the curative effect in KCZ‐ILs group was significantly better than that in the control group at weeks 2 and 3 (*p* < 0.05), meaning KCZ‐ILs exerted a more powerful effect. Further analysis on each sign showed that significant declines (*p* < 0.0001) of the severity were observed in lesion area, scaling, keratinization, and pruritus for KCZ‐ILs, as well as scaling and pruritus for Daktarin®. It reveals that the reason for the superiority on TSS improvement of KCZ‐ILs over Daktarin® may lie in the alleviation of lesion area and keratinization.

Mycological cure was generally defined as negative potassium hydroxide (KOH) examination findings and negative culture results.^31^, ^32^ However, the results of KOH examination and culture tests were found to be highly correlated. In fact, the majority of negative results in one test were related to the same negative findings in the other.^32^ Therefore, KOH examination was used for representing mycological results. Both formulations reduced the number of positive mycological patients, so that at the end of treatment, 76.47% in the KCZ‐ILs group and 56.25% in the control group were negative. When plotted versus time, there was an impressive upward trend during the treatment period of consecutive 4 weeks in the percentage of KCZ‐ILs subjects who were mycological negative, whereas the control response was almost flat.

Overall efficacy was finally evaluated according to a four‐grade scale (cured, markedly effective, effective, and ineffective) derived from TSS changes and mycological results.^24^, ^31^ As the primary efficacy variable, treatment success was defined as negative mycological result with at least 60% clinical improvement. As expected, significant differences favored KCZ‐ILs over Daktarin® during the treatment period. The stronger action of KCZ‐ILs may be due to enhanced permeation leading to persistence of therapeutic levels in skin tissues.^27^

Relapse is a result of the re‐appearance of the same original strain, which is easy to occur in many diseases and can be influenced by detection methods of post‐treatment testing.^33^, ^34^, ^35^ The detection rate for strains was reported to differ widely between polymerase chain reaction and culture.^35^ Thus, patients that appear to be cured using culture or simple mycological test could actually be harboring residual fungi. Throughout the trial period, KCZ‐ILs induced a significantly lower recurrence rate (52.94%) than in control patients (68.75%). Therefore, KCZ‐ILs were more effective in fungal clearance and preventing fungal relapse than Daktarin®.

In terms of safety, one patient in the control group experienced transient mild erythema, but none discontinued the treatment. Meanwhile, KCZ‐ILs were found to be safe and well‐tolerated.

The limitations of this study include the small sample size and short follow‐up period. Thus, long‐term maintenance of efficacy and long‐term adverse events could not be assessed.

## 4 CONCLUSION

With reference to phase 2 trials, this study was designed to be a randomized controlled clinical trial with a small sample size, assessing the efficacy and safety of a new ILs‐based formulation named KCZ‐ILs. The results showed that KCZ‐ILs (KCZ, 4.72 mg/g) showed significant superiority than Daktarin® (KCZ, 20 mg/g) in both clinical improvement and relapse prevention. Besides, KCZ‐ILs were very well‐tolerated, without any adverse event while one patient in the control group reported transient mild erythema. In conclusion, ILs loaded only 1/4 KCZ dose of Daktarin® showed a better efficacy and safety profile in the management of tinea pedis, which creates an effective pharmaceutical formulation for the treatment of skin diseases caused by fungal infection and is worthy of clinical application.

## 5 MATERIALS AND METHODS

### 5.1 Preparation, characterization, stability, and dosing of formulations

KCZ‐ILs were specifically KCZ‐loaded [Ch][Ger] aqueous solution where [Ch][Ger] was synthesized by choline bicarbonate and geranic acid at a molar ratio of 1:1. Firstly, a mixture of [Ch][Ger]/Tween‐80/glycerin/KCZ was prepared in the way reported previously.^21^ Secondly, it was heated at 90°C for 1 h till clarified, then placed overnight and centrifuged (10,000 rpm, 5 min) to obtain the supernatant. KCZ content was determined as 18.86 mg/mL by HPLC. Thirdly, the supernatant was diluted with water at a volume ratio of 1:3 to obtain 1/4 aqueous solution (KCZ, 4.72 mg/g) which is exactly KCZ‐ILs used in this study.

KCZ‐ILs were characterized by FT‐IR spectra and DSL. The stability of KCZ‐ILs in brown glass bottles was evaluated at room temperature over a 14‐month period. The representative components (KCZ and geranic acid) in KCZ‐ILs were monitored and the degradation percentage of KCZ was determined using HPLC.

Dosing of formulations was determined by the in vitro permeation test. Specifically, the porcine skin samples, harvested from 2 weeks old domestic pig, were mounted onto standard Franz cells. The cells had a contact area of 1.77 cm^2^ and a receptor volume of 7.8 ml. Phosphate buffered saline (pH 7.4) containing 20% ethonol (v/v) was used as the receptor medium, which was stirred at 300 rpm, 37°C. Test and control formulations of 500 μg were applied to the stratum corneum side of the skin samples for 6 h, respectively. To extract as much of KCZ as possible, the skin was washed and horizontally cut into pieces (20 μm). The layers of one skin sample were collected in a tube containing 480 μl of methanol and 20 μl of the internal standard bifonazole (504 μg/ml), followed by ultrasonic for 30 min and centrifuged at 20,000 rpm for 5 min. The amount of KCZ in the supernatant was measured using HPLC.

### 5.2 Study design

This randomized clinical trial was conducted at Shanghai skin disease hospital in China from August 1, 2021 to July 31, 2022. It is approved by the institutional ethics committee and registered on the Chinese Clinical Trials Registry (ChiCTR2000035486). Given the treatment success rate by week 4 of 90% for KCZ‐ILs and 41% for Daktarin® in the pre‐experiment, 36 patients (18 in each arm) were required, to achieve 90% power with an alpha of 0.05, accounting for 20% dropout. A total of 39 consecutive patients diagnosed with tinea pedis were screened and 36 satisfying inclusion and exclusion criteria were enrolled. Random number sequence was generated on computer by a simple randomization schedule. Each randomization number was sealed in an opaque envelope with a serial number on it. After signed a written informed consent, the patient with a known serial number opened the corresponding envelope to be informed of the assignment. All the patients were randomized (1:1) to receive either KCZ‐ILs or Daktarin®. Clinic visits occurred on week 0 (baseline), during weeks 1, 2, 3, and 4 (the end of the treatment period), and at week 8 (4 weeks after intervention). Compliance was assessed via the daily patient diary and the dosage was monitored by means of formulations weighing. Patients applying 80 ~ 120% of the expected doses at required time were considered to be compliant. To reduce the risk of bias, the dermatologists, mycelial examiners, data collectors, and statisticians were blinded to the information of patients until the data were locked. Changes in TSS, mycological eradication rates, and adverse effects were recorded at each visit.

### 5.3 Diagnosis, inclusion and exclusion criteria

With reference to the 2017 edition of Guidelines for the Diagnosis and Treatment of Tinea Manuum and Tinea Pedis, tinea pedis is clinically classified into blistering, interdigital erosion, and scaly keratosis based on the morphology of the lesions. Patients aged between 20 and 65 years, with any type of tinea pedis and positive fungal microscopy at the lesion site, as well as good consciousness were included. Patients were excluded if they were receiving any medication within 1 month of screening, allergic to the drugs in this study, or pregnant/lactating women. Those who withdrew in the middle of the study, or changed drugs without authorization were also excluded.

### 5.4 Formulations and treatment

KCZ‐ILs (KCZ, 4.72 mg/g) prepared as described above was used in the experiment group while the commercial Daktarin® (KCZ, 20 mg/g; Xi'an Janssen Pharmaceutical Co., Ltd.; State Drug Quantifier H20043171) was adopted in the control group. After cleaning the identified lesions, the patients topically self‐applied the test product using a cotton swab applicator, where the applied amount was able to make the lesions be covered with a thin layer of medication. The product was applied topically twice daily for 4 weeks.

### 5.5 Assessments

The primary efficacy outcome was the proportion of treatment success responders,^32^ defined as patients achieving negative mycological result and ≥60% relative reduction in TSS from baseline at week 4. Assessments including clinical TSS records and mycological analysis of skin samples, were performed at weeks 0 (baseline), 1, 2, 3, 4, and 8 (endpoint). All the scored lesions on the target foot were evaluated using the criteria incorporating seven signs and symptoms (lesion size, maceration degree, erosion area, exudation degree, scale, keratosis, and pruritus) directly attributable to tinea pedis (Table **S1**). The severity of each symptom was stratified into four levels (0 = absence of sign to 3 = worst severity of sign).^30^ Skin scraping smears were collected from the infected sites and submitted to mycological testing where KOH examination was used for representing mycological results. As the primary efficacy variable, overall efficacy was divided into four‐grade scale including cured, markedly effective, effective, and ineffective. Among these categories, “Cured” and “Markedly effective” were identified as final clinical efficient or treatment successful.^24^

Secondary outcomes mainly for evaluating the relapse of disease included the proportion of treatment success individuals at week 8 and fungal recurrence rate at weeks 2, 3, 4, and 8. Besides, TSS changes, mycological eradication rates, and the compliance at each visit were analyzed. Safety was assessed by physical examination, vital signs, and adverse events, especially irritation based on erythema, peeling, papules, and blisters.

### 5.6 Statistical analysis

Efficacy analyses were based on the per‐protocol population, which comprised the patients who had completed the entire trial as prescribed after randomization. All quantitative variables are represented as mean and standard deviation (SD). The means were compared by Student's *t*‐test for normally distributed data and Mann–Whitney test for skewed/ordinal data. Qualitative or categorical variables were described by case number and proportions No. (%). Proportions were compared via Chi‐square, McNemar or Fisher's exact test, whichever was the most applicable. All statistical tests were two‐sided and performed by Statistical Package for Social Sciences (IBM SPSS Statistics 26) at a significance level of *p* < 0.05.

## AUTHOR CONTRIBUTIONS

**Xiying Wu:** Data curation (equal); funding acquisition (lead); project administration (lead); supervision (equal); validation (equal); writing – original draft (lead); writing – review and editing (equal). **Min Shen:** Data curation (equal); project administration (equal); resources (equal). **Huan Wang:** Data curation (equal); funding acquisition (equal); writing – original draft (equal); writing – review and editing (equal). **Xue He:** Data curation (equal); project administration (equal). **Jingwen Tan:** Methodology (equal); project administration (equal). **Ruiping Wang:** Methodology (supporting). **Lianjuan Yang:** Conceptualization (supporting); project administration (supporting). **Hong Yang:** Project administration (supporting). **Jianping Qi:** Conceptualization (equal); writing – review and editing (lead). **Zhongjian Chen:** Conceptualization (equal); resources (lead); supervision (equal). **Quangang Zhu:** Conceptualization (equal); funding acquisition (equal); supervision (lead); writing – review and editing (equal).

## CONFLICT OF INTEREST

The authors have no conflicts of interest relevant to this article.

### PEER REVIEW

The peer review history for this article is available at https://publons.com/publon/10.1002/btm2.10463.

## Supporting information


**Appendix S1.** Supporting Information.

## Data Availability

The data that support the findings of this study are available from the corresponding author upon reasonable request.
