# Supplementary material for: Evaluation of the efficacy and safety of ionic liquids containing ketoconazole in patients with tinea pedis: A randomized controlled clinical trial
Source: Bioeng Transl Med. 2022 Dec 2;8(3):e10463. doi: 10.1002/btm2.10463 (PMC10189433; doi:10.1002/btm2.10463)
Supplement: Supplementary file 1 — Appendix S1. Supporting Information. [file BTM2-8-e10463-s001.docx]

**Supporting Information**

**Evaluation of the Efficacy and Safety of Ionic Liquids Containing Ketoconazole in Patients with Tinea Pedis：A Randomized Controlled Clinical Trial**

Xiying Wu^1,2,†^ · Min Shen^1,†^ ··Huan Wang^3,†^ ··Xue He^1^ · Jingwen Tan^1^ ·Ruiping Wang^1^ · Lianjuan Yang^1^ ·Hong Yang^1^ · Jianping Qi^1,2,*^ · Zhongjian Chen^1,*^ · Quangang Zhu^1,*^

^1^ Shanghai Skin Disease Hospital, Tongji University School of Medicine, Shanghai, China

^2^ School of Pharmacy, Fudan University, Shanghai, China

^3^ School of Pharmacy, Naval Medical University, Shanghai, China

† Xiying Wu, Min Shen and Huan Wang contributed equally to this study and are joint first authors.

* Jianping Qi, Zhongjian Chen and Quangang Zhu contributed equally to the study and are **Correspondence**

Jianping Qi, School of Pharmacy, Fudan University, Shanghai 201203, China

Email: qijianping@fudan.edu.cn

Zhongjian Chen, Shanghai Skin Disease Hospital, Tongji University School of Medicine, Shanghai 200443, China

Email: [aajian818@sina.com](mailto:aajian818@sina.com)

Quangang Zhu, Shanghai Skin Disease Hospital, Tongji University School of Medicine, Shanghai 200443, China

Email: [qgzhu@126.com](mailto:qgzhu@126.com)

**Fig. S1** The chemical structures of KCZ and [Ch][Ger] in KCZ-ILs


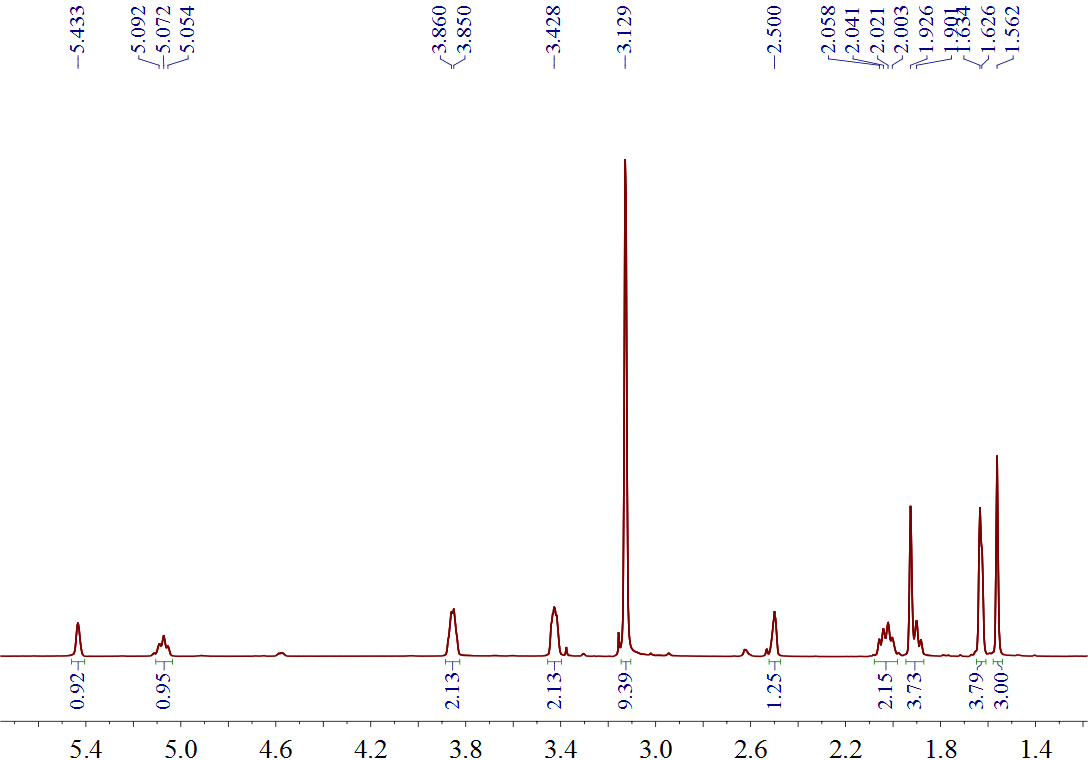


**Fig. S2** ^1^H**-**NMR spectra of [Ch][Ger] in DMSO-*d_6_*. ^1^H-NMR was performe on Bruker Avance III 400 MHz spectrometer (Berne, Switzerland).


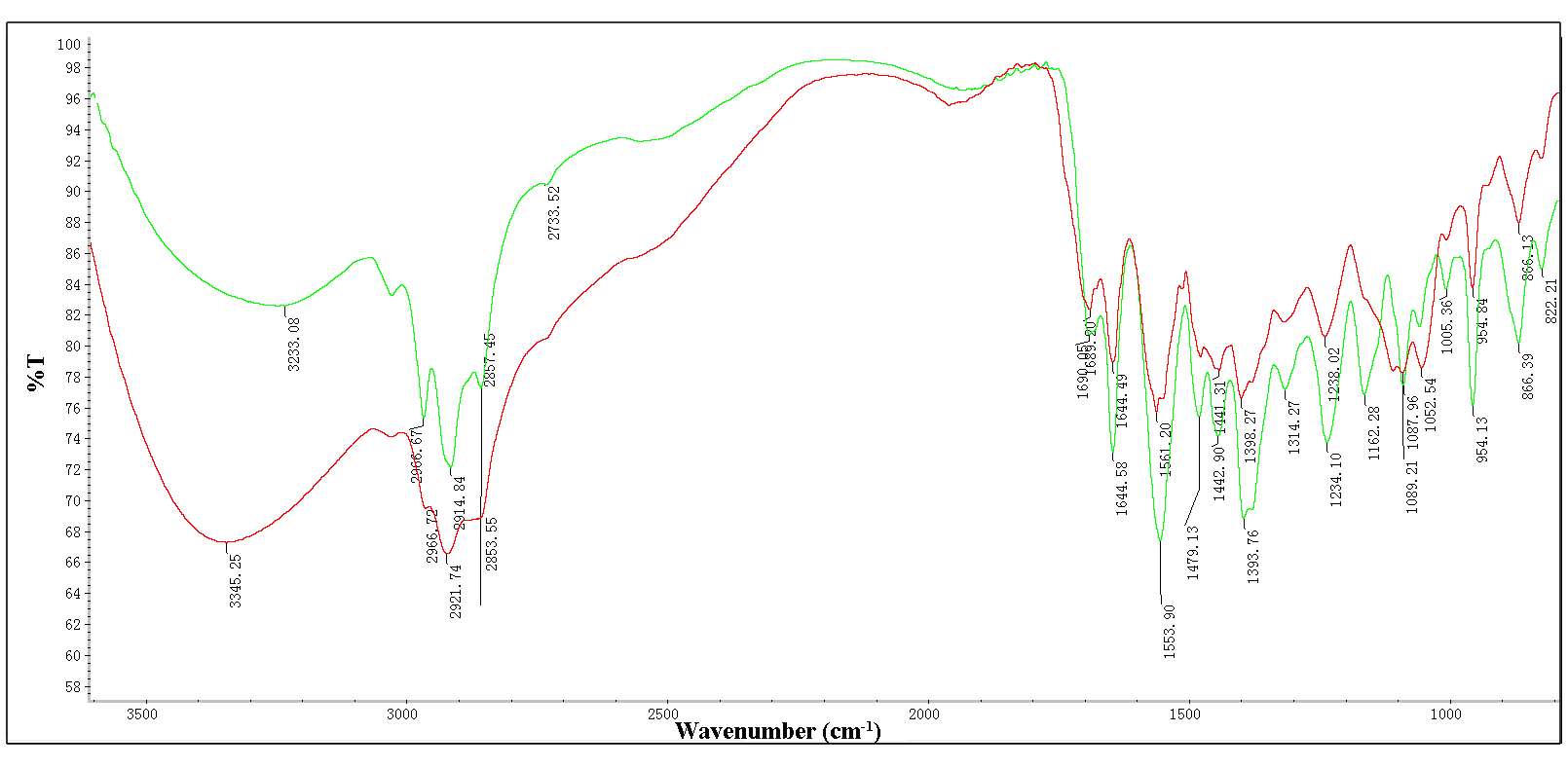


**Fig. S3** FT-IR spectra of [Ch][Ger] (green) and KCZ-ILs (red). FT-IR Spectra were carried out with potassium bromide-disk on Thermo Scientific Nicolet FT-IR spectrometer (WI, USA).


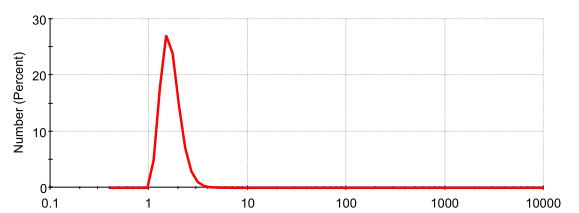


**Fig. S4** Size and size distribution of KCZ-ILs at 25℃. DSL was performed on Malvern Zetasizer Nano-ZS (Worcester, UK).

**
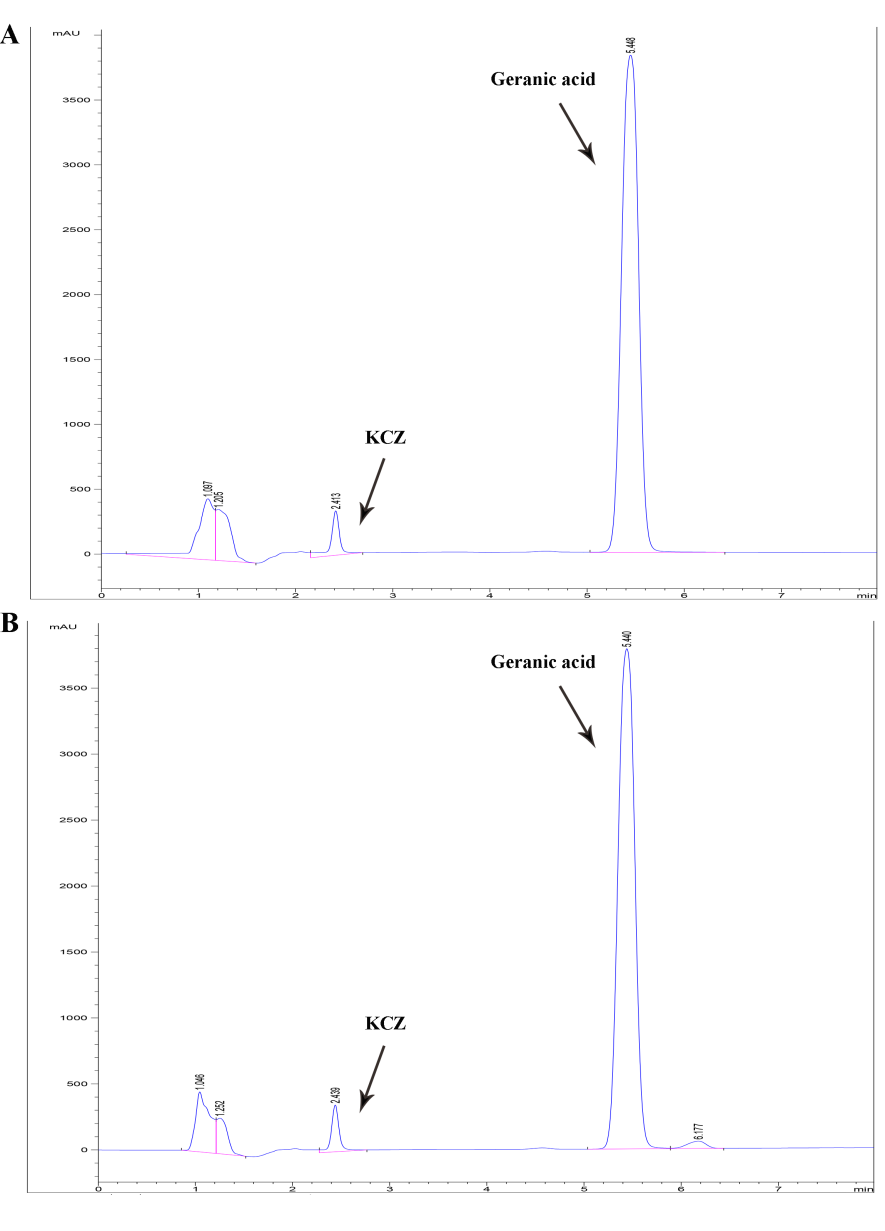
**

**Fig. S5** HPLC analysis for the KCZ-ILs. (A) freshly prepared KCZ-ILs; (B) KCZ-ILs stored in at room temperature after 14 months. HPLC was performed on an Agilent Technologies 1200 HPLC system (Agilent, Santa Clara, USA) with Agilent Eclipse XDB-C18 column (5 *μ*m, 4.6 mm×150 mm). KCZ and geranic acid were monitored at 235 nm, and the mobile phase was set as sodium dihydrogen phosphate buffer/acetonitrile (52:48, v/v) at a flow rate of 1.0 mL/min.


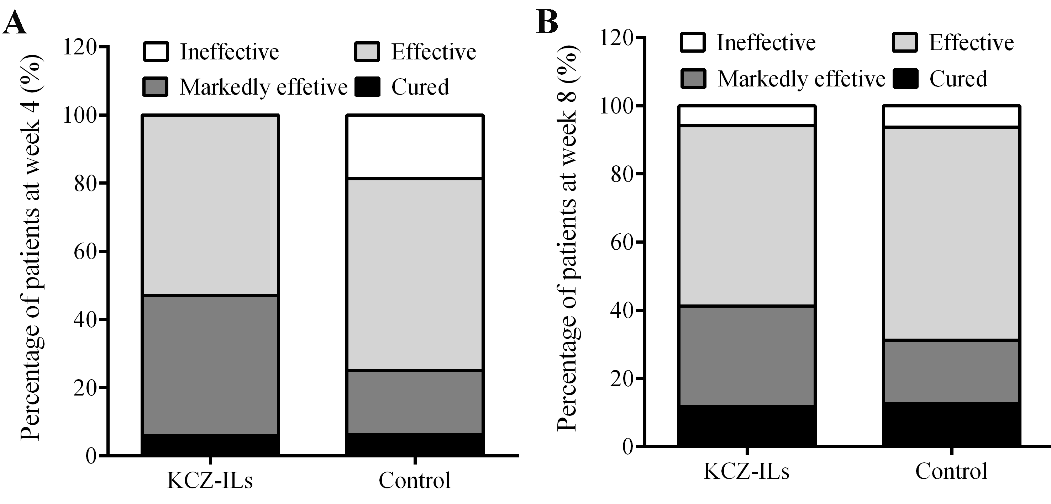


**Fig. S6** Overall efficacy in tinea pedis patients. Data are shown for weeks 4 (A) and 8 (B, 4 weeks post-treatment).

**Table S1** Clinical scoring criteria

| **Signs** | **0** | **1** (Mild) | **2** (Moderate) | **3** (Severe) |
| --- | --- | --- | --- | --- |
| **Lesion** | abscent | < 2.5 cm^2^ | 2.5~5 cm^2^ | > 5 cm^2^ |
| **Maceration** |  | 1 toe seam | 2 toe seams | 3 toe seams |
| **Erosion** |  | < 2.5 cm^2^ | 2.5~5 cm^2^ | > 5 cm^2^ |
| **Exudation** |  | superficial | concomitant scabs | concomitant fluid |
| **Scale** |  | < 2.5 cm^2^ | 2.5~5 cm^2^ | > 5 cm^2^ |
| **Keratosis** |  | < 2.5 cm^2^ | 2.5~5 cm^2^ | > 5 cm^2^ |
| **Pruritus** |  | occasional | often | continued |

**Table S2** Comparison of TSS [mean (SD)] between KCZ-ILs and control groups *^a^*

| **Time points** | **KCZ-ILs** | **Control** | **t** | **P** |
| --- | --- | --- | --- | --- |
| Baseline | 8.76 (2.14) | 9.44 (3.18) | -0.717 | 0.479 |
| Week 1 | 6.41 (2.21) | 8.00 (2.73) | -1.841 | 0.075 |
| Week 2 | 4.88 (2.00) | 6.94 (2.17) | -2.831 | 0.008* |
| Week 3 | 4.35 (1.73) | 6.06 (2.93) | -2.055 | 0.048* |
| Week 4 | 3.82 (2.13) | 5.00 (2.76) | -1.377 | 0.178 |
| Week 8 | 3.88 (2.20) | 4.88 (3.05) | -1.076 | 0.290 |

*^a^* Repeated measure variance analysis showed that the difference between two groups was significant (F = 58.58, P < 0.001), and t-test was further performed.

**Table S3** Comparison of the scores in each sign at baseline and week 4 (mean ± SD)

| **Signs** | **KCZ-ILs (n=17)** | | **t** | **P** | **Control (n=16)** | | **t** | **P** |
| --- | --- | --- | --- | --- | --- | --- | --- | --- |
|  | **Baseline** | **Week 4** |  |  | **Baseline** | **Week 4** |  |  |
| Lesion | 2.24 ± 0.75 | 1.35 ± 0.79 | 4.23 | 0.001 | 2.13 ± 0.81 | 1.53 ± 0.83 | 2.78 | 0.015 |
| Maceration | 0.29 ± 0.59 | 0.00 ± 0.00 | 2.06 | 0.056 | 0.75 ± 1.13 | 0.13 ± 0.52 | 2.36 | 0.033 |
| Erosion | 0.12 ± 0.33 | 0.00 ± 0.00 | 1.46 | 0.163 | 0.31 ± 0.70 | 0.00 ± 0.00 | 1.47 | 0.164 |
| Exudation | 0.06 ± 0.24 | 0.00 ± 0.00 | 1.000 | 0.332 | 0.13 ± 0.34 | 0.00 ± 0.00 | 1.47 | 0.164 |
| Scale | 2.12 ± 0.78 | 1.29 ± 0.69 | 3.85 | 0.001 | 2.06 ± 0.77 | 1.33 ± 0.82 | 3.16 | 0.007 |
| Keratosis | 2.12 ± 0.70 | 1.12 ± 0.86 | 4.12 | 0.001 | 1.88 ± 0.96 | 1.33 ± 0.72 | 2.82 | 0.014 |
| Pruritus | 1.82 ± 0.88 | 0.06 ± 0.24 | 8.75 | <0.001 | 2.19 ± 0.75 | 0.40 ± 0.51 | 11.31 | <0.001 |

**Table S4** Comparison of the scores in each sign at baseline and week 8 (mean ± SD)

| **Signs** | **KCZ-ILs (n=17)** | | **t** | **P** | **Control (n=16)** | | **t** | **P** |
| --- | --- | --- | --- | --- | --- | --- | --- | --- |
|  | **Baseline** | **Week 8** |  |  | **Baseline** | **Week 8** |  |  |
| Lesion | 2.24 ± 0.75 | 1.35 ± 0.86 | 4.24 | 0.001 | 2.13 ± 0.81 | 1.56 ± 0.96 | 2.76 | 0.014 |
| Maceration | 0.29 ± 0.59 | 0.00 ± 0.00 | 2.06 | 0.056 | 0.75 ± 1.13 | 0.13 ± 0.50 | 2.61 | 0.020 |
| Erosion | 0.12 ± 0.33 | 0.00 ± 0.00 | 1.46 | 0.163 | 0.31 ± 0.70 | 0.00 ± 0.00 | 1.78 | 0.096 |
| Exudation | 0.06 ± 0.24 | 0.00 ± 0.00 | 1.000 | 0.332 | 0.13 ± 0.34 | 0.00 ± 0.00 | 1.46 | 0.164 |
| Scale | 2.12 ± 0.78 | 1.29 ± 0.77 | 3.85 | 0.001 | 2.06 ± 0.77 | 1.25 ± 0.68 | 4.96 | <0.001 |
| Keratosis | 2.12 ± 0.70 | 1.18 ± 0.64 | 5.89 | <0.001 | 1.88 ± 0.96 | 1.44 ± 0.89 | 2.15 | 0.048 |
| Pruritus | 1.82 ± 0.88 | 0.06 ± 0.24 | 8.75 | <0.001 | 2.19 ± 0.75 | 0.50 ± 0.82 | 7.13 | <0.001 |
